# Advances in genetic diagnosis and therapy of hereditary heart disease: a bibliometric review from 2004 to 2024

**DOI:** 10.3389/fmed.2024.1507313

**Published:** 2025-01-08

**Authors:** Huixi Ma, Yun Wang, Yang Jia, Linjun Xie, Lini Liu, Dingyi Zhang, Xinyue Ma, Yingkun Guo, Rong Xu

**Affiliations:** ^1^Key Laboratory of Obstetric and Gynecologic and Pediatric Diseases and Birth Defects of Ministry of Education, Department of Radiology, West China Second University Hospital, Sichuan University, Chengdu, China; ^2^West China Medical School, Sichuan University, Chengdu, China

**Keywords:** hereditary heart disease, cardiac death, genetic diagnostic, cardiomyopathy, scientometrics

## Abstract

Hereditary heart disease (HHD) is a series of cardiac disorders associated with monogenic or polygenic abnormalities and is one of the leading causes of sudden death, particularly in young adults. The updated European Cardiology guideline for cardiomyopathies provides the first comprehensive summary of genotyping, imaging, and therapy recommendations for inherited cardiomyopathies, but still lacks a comprehensive discussion of research advances and future trends in genetic diagnosis and therapy of HHD. Our research aims to fill this gap. Bibliometric analysis software (CiteSpace 6.3.R1, VOSviewer 1.6.18, and Scimago Graphica) was used to analyze the general information, trends, and emerging foci of HHD in the past 20 years, including author, country, institution, keyword, and so on. There were 5,757 publications were screened and aggregated in the database, including 1876 reviews and 3,881 articles. Hypertrophic cardiomyopathy (HCM), arrhythmogenic cardiomyopathy (ACM), Brugada syndrome (BrS), myocardial amyloidosis, and Fabry disease (FD) were the main types of HHD that were explored in greater depth. Moreover, new diagnostic methods, clinical cohorts, and genetically targeted therapies for HHD patients are key research hotspots. The relationship between the pathogenicity of genes and prognosis will become increasingly important for therapy.

## 1 Introduction

Hereditary heart disease (HHD) is a type of cardiomyopathy linked to monogenic or polygenic abnormalities and is a leading cause of sudden death (1). The different types of genes may be presented with different cardiomyopathic phenotypes and symptoms, or several types of genes manifest with similar phenotypes (2, 3). Hypertrophic cardiomyopathy (HCM), dilated cardiomyopathy (DCM), arrhythmogenic right ventricular cardiomyopathy (ARVC), and restrictive cardiomyopathy (RCM) are the most common hereditary cardiomyopathy. Recently, non-dilated left ventricular cardiomyopathy (NDLVC) is a new disease category that is defined as the presence of non-ischemic LV scarring or fatty replacement regardless of the presence of global or regional wall motion abnormalities, or isolated global LV hypokinesia without scarring. In addition, there were experts have suggested that metabolic abnormalities such as Fabry disease (FD) may be associated with cardiac infarction. The continuous updating of diagnosis and treatment guidelines contributes to the development of cardiovascular disease (4–6).

Genetic diagnosis is crucial for a definitive diagnosis of HHD due to its heterogeneity and complexity. Genetic testing has advanced quickly as a result of the Precision Medicine Initiative in 2015, and it is already a common element of clinical management (7). Over 100 genes are potentially associated with cardiomyopathies. The 2023 European Cardiology guideline provides a comprehensive summary of genotyping, imaging, and therapy recommendations for inherited cardiomyopathies (8). It emphasizes the importance of familial testing and standardized management for genetic-phenotypically positive patients. Nevertheless, many clinical studies of HHD lack trustworthy evidence, and bibliometric assessments that thoroughly examine the worldwide distribution of HHD research are scarce (9–11).

This study aims to fill a gap in the literature by providing a comprehensive analysis of genetic diagnostic and therapy research in HHD between 2004 and 2024 using bibliometric and visualization analyses. Bibliometrics helps extract influence and develop trends in published research findings by providing quantitative information on country, institution, author, and journal distribution (12). This analysis may help identify hot spots and frontiers in HHD research, enhancing understanding of research centers, regions, and hotspots.

## 2 Materials and methods

**Figure 1:**
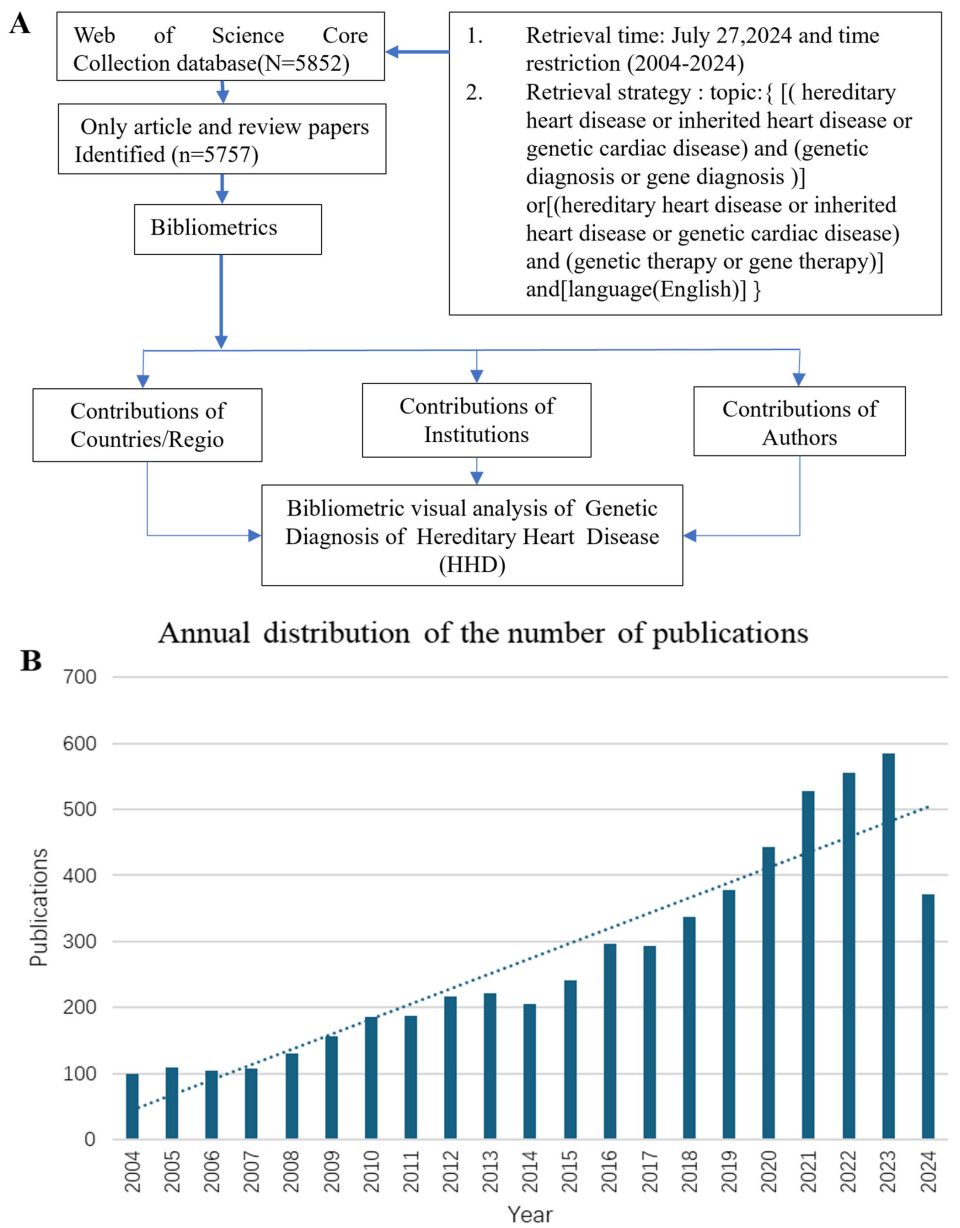
Flow chart of literature retrieval of genetic diagnosis and therapy of HHD research **(A)**. Global trend chart of annual publications and citations of genetic diagnosis of HHD related to research from 2004 to 2024 **(B)**.

The study used the Web of Science Core Collection (WoSCC) database to search for publications with the following search strategy: Topic = {[(hereditary heart disease or inherited heart disease or genetic cardiac disease) and (genetic diagnosis or genetic diagnostics or gene diagnosis)) or ((hereditary heart disease or inherited heart disease or genetic cardiac disease) and (genetic therapy or gene therapy)]} and language = (English) and article type = (articles or reviews) and time span = (January 2004 to December 2024) (Figure 1A). Incomplete or unpublished literature was excluded, and 5,757 results were included. The titles, authors, abstracts, keywords, and cited references were then imported into the scientific knowledge graph analysis tools. The study used CiteSpace 6.3.R1, VOSviewer 1.6.18, and Scimago Graphica to analyze literature, removing duplicates and presenting results as a visual map (13–15). CiteSpace 6.3.R1 was used for visual analysis of countries/regions and institutions, co-citation analysis of references, burst keyword detection, and clustering analysis of keywords. VOSviewer 1.6.18 was used for the visual analysis of authors and co-cited authors, and Scimago Graphica was used to visualize the global distribution of publications. The final image displayed nodes representing analyzed items, with larger nodes indicating a larger number of publications and the thickness of the line indicating the degree of co-occurrence or referencing (16, 17). Clustering is another method to analyze a research domain by reflecting a unique theme. Burst detection was used to identify sudden and significant increases in the frequency of specific features or events. It enables the detection of bursts or spikes in activity and allows us to overcome the limitation of relying solely on cumulative indicators. This approach allows researchers to identify and analyze significant events or patterns that may have a significant influence on the overall system or network under study.

## 3 Results

### 3.1 Annual distribution of the number of publications

The number of publications per year reflects the rate of development of knowledge in the discipline and is one of the most important indicators to explain trends in a field. Between 2004 and 2024, there has been a rapid increase in publications, particularly after 2017 (Figure 1B).

### 3.2 Country, institution, and co-author analysis

**Figure 2:**
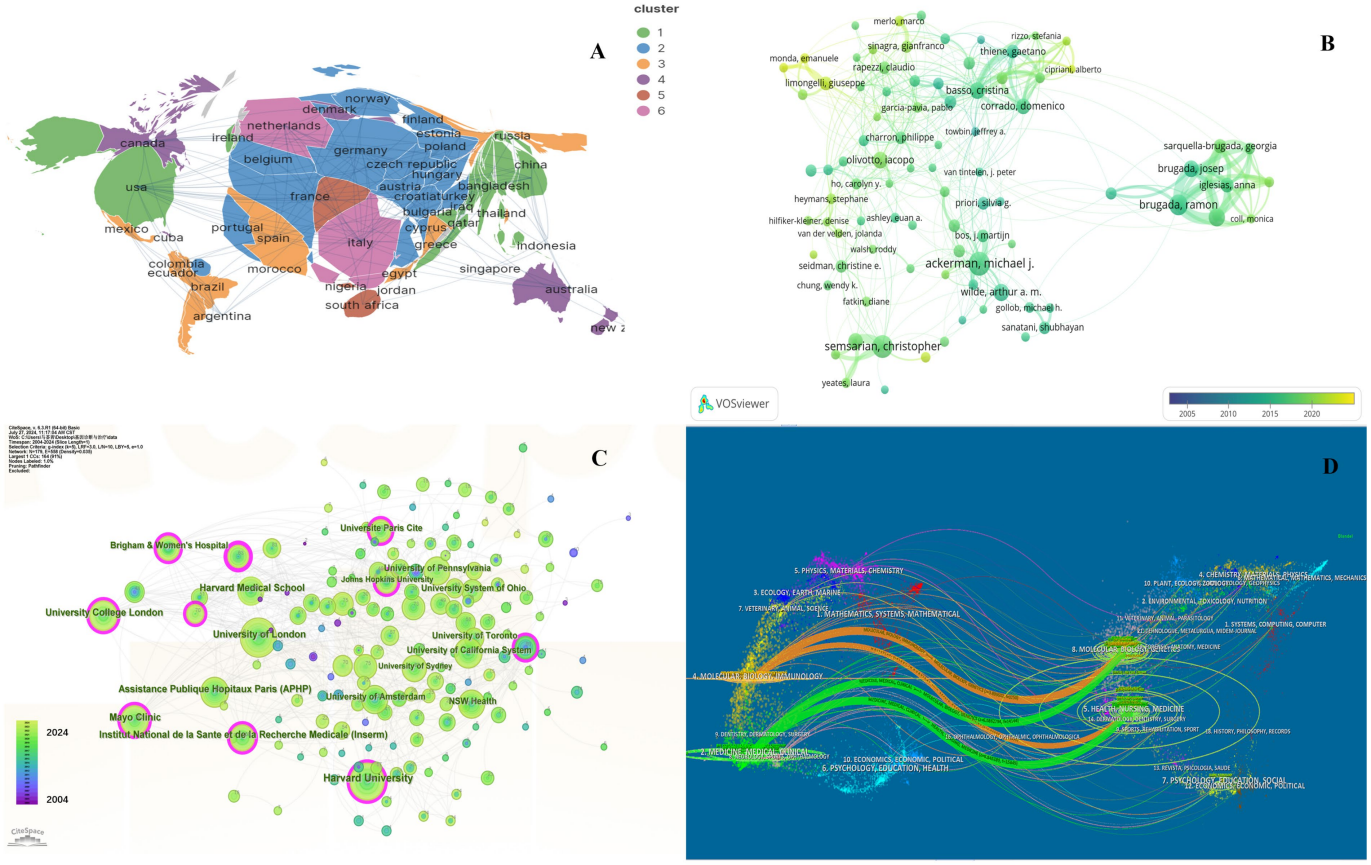
Collaboration of countries/regions, authors, institutions and journals in the field of genetic diagnosis and therapy of HHD. **(A)** Geographical map of publications. **(B)** Network map of author analysis based on VOSviewer. **(C)** Network map of institution analysis based on CiteSpace. **(D)** Dual-map overlay of journals based on CiteSpace (2004–2024).

Further details concerning nations, medical research production teams, and their collaboration can be found on the knowledge map. The United States, Italy, England, and China are recognized as the most prominent nations in the field of HHD research, exhibiting significant influence (Supplementary Table S1). Among them, the United States published a total of 2017 articles in 20 years owing to its excellent research team. The major research institutions are Harvard University, University of London, Assistance Publique Hopitaux Paris (APHP), Mayo Clinic, and University College London (Figure 2C; Supplementary Table S2).

In general, many scholars carry out research in HHD and have obtained remarkable results (Figure 2B). Show some authors have published several articles with excellent quality, such as Michael J. Ackerman, Christopher Semsarian, Ramon Brugada, and Oscar Campuzano (Table 1). All authors were ranked according to Total publications (TP) and H-indices to identify the most influential experts in the field of HHD genetic diagnosis and therapy in the last decade. In this analysis, Professor Michael J. Ackerman from Mayo Clinic was ranked first with 951 publications and an H-index of 108, followed by Professor Christopher Semsarian from the University of Sydney, who published 375 papers and had an H-index of 55.

**Table 1:** Top 10 authors of studies on genetic diagnosis and therapy of hereditary heart disease (HHD).

Rank	Author	Country	Institution	TP	ACI	H-index
1	Ackerman, Michael J.	United States	Mayo Clinic	951	42.79	108
2	Semsarian, Christopher	Sydney	Unive of Sydney	375	31.58	55
3	Brugada, Ramon	Spain	CIBER-Centro de Investigacion Biomedica-en Red	467	39.22	59
4	Campuzano, Oscar	Spain	Universitat de Girona	156	16.86	29
5	Basso, Cristina	Italy	Universitaria di Bologna	916	35.93	91
6	Wilde, Arthur A. M.	Netherland	Amsterdam Cardiovascular Sciences	959	29	140
7	Brugada, Josep	Spain	St Joan Deu Hosp Barcelona	847	48.09	91
8	Olivotto, Iacopo	Italy	Institute of Sports Medicine and Science	433	34.75	58
9	Corrado, Domenico	Italy	Università degli Studi di Padova	502	51	100
10	Calkins, Hugh	United States	Johns Hopkins University School of Medicine	913	213	141

TP, total publications; ACI, average citations per item.

### 3.3 Analysis of keywords

**Figure 3:**
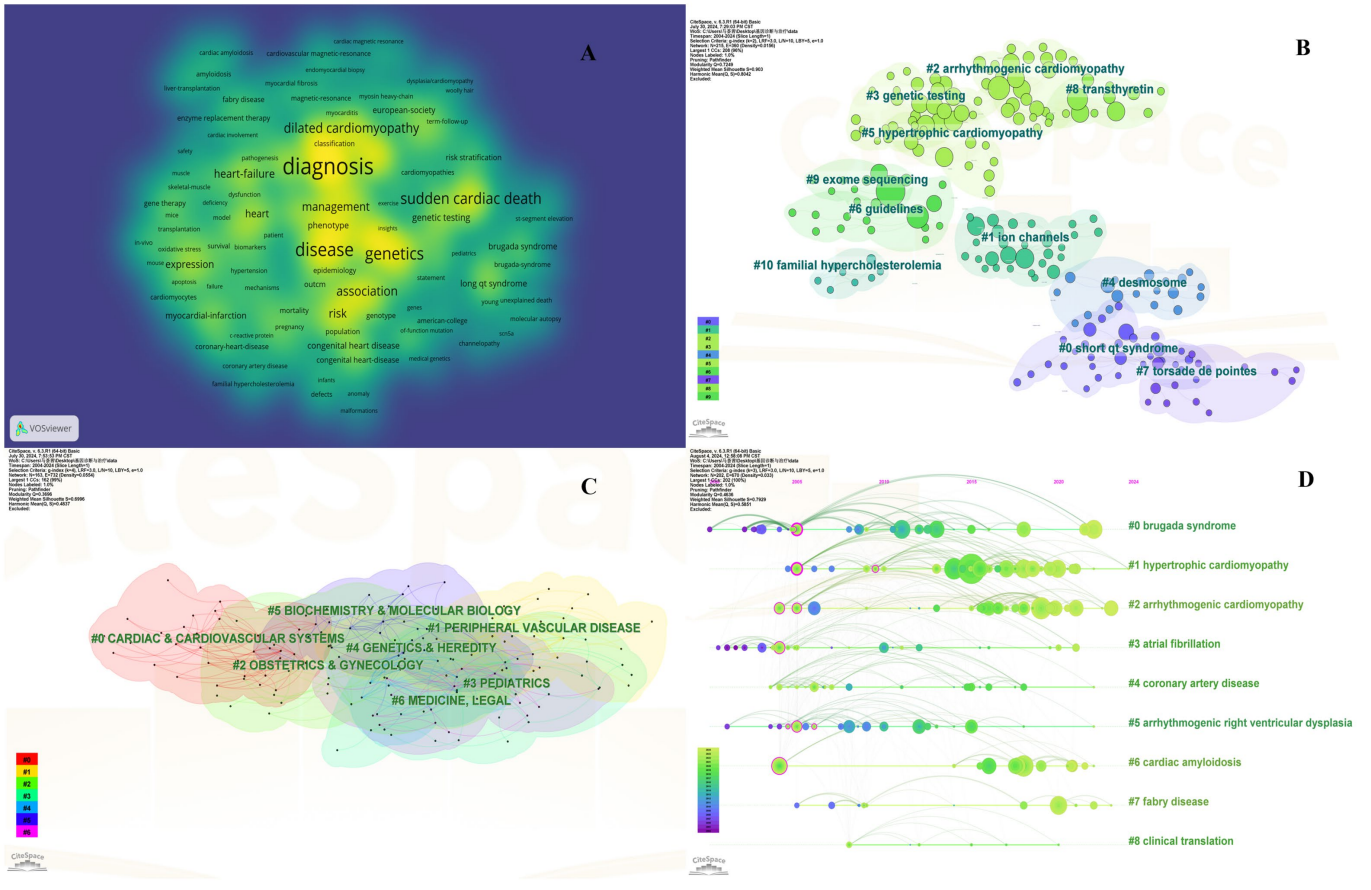
Collaboration of keywords in the field of genetic diagnosis and therapy of HHD. **(A)** Network map of keyword analysis based on VOSviewer. **(B)** Network map of the top 10 keyword cluster analyses based on CiteSpace. **(C)** Network map of the top 10 subject category analyses based on CiteSpace. **(D)** Timeline view of keywords based on CiteSpace.

Keyword investigation can explain medical research topics. Because keywords explain the central idea that summarizes and condenses the major information of the article. As illustrated in Figure 3A, the most important keywords are disease, mutations, hypertrophic cardiomyopathy, heart failure, and sudden cardiac death (SCD). To avoid many keywords obscuring the real focus of the research, we used clustering analysis of Citespace to demonstrate a classification of keywords (Figure 3B). To further refine the study and capture research hotspots, the top 11 keyword clusters were divided into 2 sections: heart disease (#0 short qt syndrome, #2arrhythmogenic cardiomyopathy, #5 hypertrophic cardiomyopathy, and #10 familial hypercholesterolemia), and diagnosis and therapy (#1 ion channels, #3 genetic testing, and #4 desmosome, #6 guidelines, #7 torsade de points, #8 transthyretin, and #9 exome sequencing).

Clustering is usually evaluated for reliability using the average silhouette value. In general a silhouette value higher than 0.7 indicates valid and convincing clustering a value between 0.5 and 0.7 indicates reasonable clustering and a value lower than 0.5 indicates unreliable clustering. We obtained clusters with silhouette values all above 0.7 suggesting the results are very convincing in this analysis.

### 3.4 Analysis of co-occurrence of subject classification

The subject classification system in the Web of Science Core Collection (WoSCC) is controversial due to its reliance on journal citation patterns and expert assessments. Therefore, we used CiteSpace for literature analysis. CiteSpace is widely used to describe the disciplinary co-occurrence features and allows the identification of associations between SC domains and HHD. This characteristic facilitates the construction of a network that demonstrates interconnections within disciplines and identifies high-impact disciplinary categories at a micro level. HHD is a typically interdisciplinary program confirmed by the disciplinary co-occurrence analysis of CiteSpace. The main relevant disciplines of HHD were cardiac and cardiovascular systems, peripheral vascular disease, obstetrics and gynecology, pediatrics, genetics and hereditary, biochemistry and molecular biology (Figure 3C).

### 3.5 Reference and co-citation journal analysis

The co-citation analysis serves to summarize the cited number and journal of reference in a published paper. The cited references reflect the foundation of research and frontiers of HHD. The top five most-cited references are important for the development of HHD (Table 2). One of the most cited references was published in the Genetics in Medicine by Sue Richards, Chair, et al., which provided a systematic overview of clinical genetic testing and sequence variation, inspiring many subsequent investigators (18). The second most cited reference was published in Heart Rhythm and was written by Towbin et al. (19). The third most cited reference was published in European Heart Journal by Elliott et al. (20). The biplot overlay of journals shows the association between journals and cited journals in the influence studies of HHD (Figure 2D). The figure shows the three primary citation pathways from clinical medicine, and genetics to bio-physiology. The analysis of clusters and key nodes in the co-citation network provides insights into the knowledge structure and development in the research of HHD. The timeline view shows the co-citation network in HHD research by years (Figure 3D). The circular nodes in the figure denote the frequency of references, and the larger nodes the higher citation frequencies. The figure shows the top 9 different research directions combined with the clustering and time-slicing techniques by visualization method, and it provides a comprehensive view of topic distribution, research trends, and interconnections over time. According to the colors of the nodes, the blue represents early literature, and the green represents more recent publications. The figures and tables above show the study of HHD has transitioned from early pathophysiology research to more comprehensive investigations focusing on specific genes and disease species. The main research disease species in recent years have been Hypertrophic cardiomyopathy (HCM), arrhythmogenic cardiomyopathy (ACM), Brugada syndrome (BrS), myocardial amyloidosis, and Fabry disease (FD).

**Table 2:** Top 5 most cited references of publications on genetic diagnosis and therapy of hereditary heart disease (HHD).

Ranking	Title	Journals	Publication year	TC	First author
1	Standards and guidelines for the interpretation of sequence variants: a joint consensus recommendation of the american college of medical genetics and genomics and the association for molecular pathology	Genetics in medicine	2015	19217	Sue Richards, Chair
2	2019 HRS expert consensus statement on evaluation, risk stratification, and management of arrhythmogenic cardiomyopathy	Heart rhythm	2019	446	Jeffrey A. Towbin
3	2014 ESC Guidelines on diagnosis and management of hypertrophic cardiomyopathy the task force for the diagnosis and management of hypertrophic cardiomyopathy of the european society of cardiology (ESC)	European heart journal	2014	3260	Perry M. Elliott
4	Tafamidis treatment for patients with transthyretin amyloid cardiomyopathy	New England journal of medicine	2018	1479	Mathew S. Maurer
5	Genotype and lifetime burden of disease in hypertrophic cardiomyopathy: insights from the sarcomeric human cardiomyopathy registry (SHaRe)	Circulation	2018	452	Carolyn Y. Ho

TC, total citations.

## 4 Discussion

### 4.1 General information

In this study, we first analyze the global research and summarize the development in the mostly concerned direction of HHD. The literature analysis in this paper shows a general upward trend in HHD research from 2004 to 2024, indicating a growing interest in genetic diagnosis and therapy of HHD. In the country analysis, the United States has published more papers on genetic diagnosis and therapy of HHD. The institutions with the most publications were Harvard University, University of London, APHP, Mayo Clinic, and University College London. In addition, the journals with the most publications were Circulation, the Journals of the American College of Cardiology, and the New England journal of medicine. Genetic diagnosis and therapy of HHD is considered a multidisciplinary subject, it shows interest not only in cardiology and genetics but also in pediatrics, biochemistry & molecular biology, and so on (21).

### 4.2 Evolution of the relationship between hereditary heart disease and genetic diagnosis

**Figure 4:**
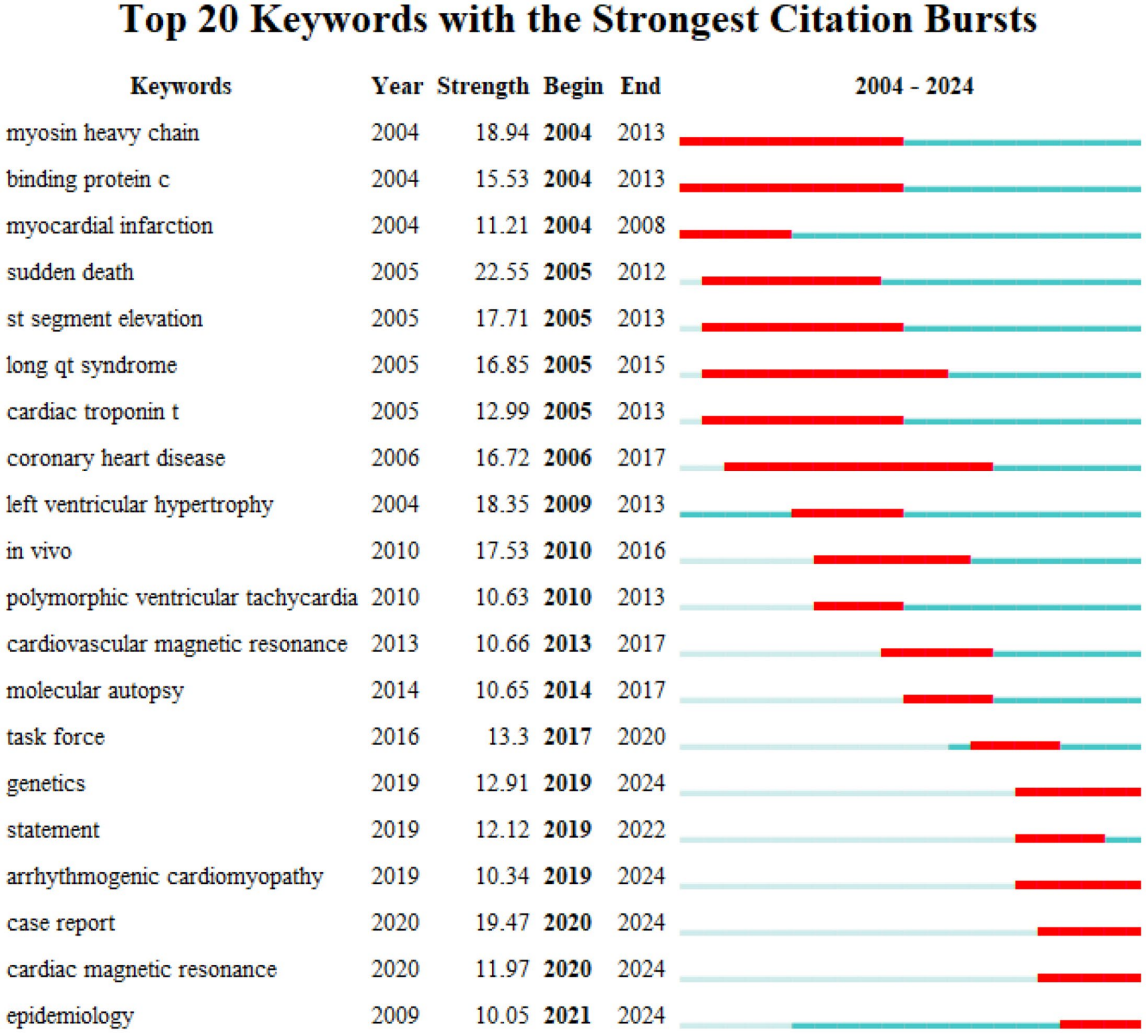
Top 20 keywords with the strongest citation bursts (2004–2024).

Analysis of the co-citation patterns of burst keywords and references showed that while earlier studies on HHD focused on pathophysiological mechanisms, later studies have focused more on the association between specific genes and the disease (22–25). This finding is consistent with the research process of major authors. According to our analysis, Michael J. Ackerman’s earlier study investigated the effects of malignant mutations in the *β*-myosin heavy chain and troponin T genes on patients with HCM, and further designed experiments to confirm the underlying mechanism of HCM caused by mutations in the myosin-binding protein-C gene (26). In recent years, Michael J. Ackerman and his team have focused on the genomics and genotype–phenotype relationships of inherited cardiovascular diseases. In addition, early studies by Professor Ramon Brugada focused on pharmacological treatment of short QT syndrome and pathological changes in inherited arrhythmias (27). More recent studies have focused on the genetic basis and therapeutic strategies of Brugada syndrome (Figure 4).

The diagnosis of HHD is highly correlated with the development of detection techniques. Genetic testing technologies have changed rapidly over the past few years as flourished genomics and advanced precision medicine (28, 29). Next-generation sequencing (NGS) is the prime method of clinical genetic testing, which not only improves the efficiency and speed of gene sequencing, but also greatly reduces the cost of clinical genetic testing. This approach is very important in case studies of dilated cardiomyopathy with uncertain family history (30). The use of NGS technology in clinical diagnostics has spawned a variety of molecular diagnostic tests, including single-gene assays, gene panel tests, enrichment scoring (ES), and genomic selection (GS) (31). Therefore, it is a challenge for clinicians to select the most appropriate test method for patients with suspected genetic diseases is currently.

Genome-wide association studies (GWAS) are another widely used genetic diagnostic tool that enables high-throughput analysis of large-scale gene sequence data, resulting in more information in a shorter period (32). Rasooly et al. (33) identified 39 genetic abnormalities associated with heart failure by performing GWAS analysis on nearly 100,000 patients, which may provide new targets for clinical treatment. However, GWAS identifies only a few genetic susceptibility loci in each study typically, and not all genetic disorders significantly differ across populations. And GWAS has limited heritability that can be explained. In some studies of HHD, its results have been unsatisfactory, especially in complex heart failure (34–38).

Therefore, clarification of specific gene mutations and optimized detection techniques are important to the clinical management of HHD. This research trend fits well with the keywords such as ‘statement’, ‘working group’, and ‘epidemiology’, which appeared most frequently in the keyword analysis. These keywords emphasize the importance of clinical management in HHD patients. Meanwhile, HHD-related genetic testing has been gradually extended to family members. According to the current expert consensus, family members should be screened for genetic cascades if have a detected mutation (39–42). For some specific familial mutations, current guidelines recommend annual clinical follow-up with electrocardiography and echocardiography for family members with positive mutations (43–48).

### 4.3 Research hotspots

In our study, the keyword and timeline clustering showed recent research hotspots are arrhythmogenic cardiomyopathy (ACM), hypertrophic cardiomyopathy (HCM), Brugada syndrome (BrS), Fabry disease (FD), and cardiac amyloidosis (49–53). We have summarized the latest literature for these categories of diseases.

#### 4.3.1 Arrhythmogenic cardiomyopathy (ACM)

ACM is the most common cause of SCD in young people. The most frequently inherited form of ACM is caused by mutations in the pachytene-plakophilin-2 (PKP2) gene. Noteworthy, recent findings by Garcia-Quintans Nieves and Sacristán Soriano, et al. suggest that activation of MYH10 corrects the deleterious effects of PKP2 mutants on cardiac contraction, which has potential clinical implications for ACM therapy (54). In ACM patients, the less pathogenic variants (PVs) in different sets of genes, cause the slight disease phenotype (55, 56). And the multiple PVs in arrhythmias and heart failure patients always had a poorer prognosis.

Especially arrhythmogenic left ventricular cardiomyopathy (ALVC) patients are likely to combine with PLN and DSP variants and are associated with a high risk of heart failure. In patients initially diagnosed with DCM, the DSP variant predicted an increased risk of malignant arrhythmia and sudden death with preserved left ventricular ejection fraction. Gene variants, gender, age, and other environments may affect prognosis in ACM. Some studies reported the Newfoundland TMEM43 S358L gene variant in males being associated with very high permeability and arrhythmia risk rather than in females (57). And e PLN R14del gene in females usually has a poorer prognosis than males (58). However, age also affects ACM, and screening according to age is recommended by the ACM recommendations. Future research on genetic and environmental modifiers is expected to enhance personalized risk prediction and screening strategies (59, 60).

#### 4.3.2 Hypertrophic cardiomyopathy (HCM)

As the most common genetic disease of the heart, HCM is primarily characterized by left ventricular hypertrophy (61). Mutations in the genes encoding the components of myofilament proteins are the primary cause of HCM. Mutations in the MYH7 and MYBPC3 genes account for 40% of all HCM cases (62, 63), and individuals with double allele mutations in MYBPC3 develop a more HCM, in which the patient is predisposed to hypertrophy, severe systolic and diastolic dysfunction, progressive HF, and death within 1 year. The results of a large multicenter cohort study suggest that the presence of sarcomere-negative mutations is associated with early onset and may be a strong predictor of adverse clinical outcomes, including ventricular arrhythmias and HF (64). These results underscore the importance of genotype in prognosis evaluation and clinical management guidance of HCM patients. In addition, preclinical studies on novel precision therapies for gene editing had more attention in recent years (65–68). Further treatment can utilize virus-mediated gene replacement therapy or mutation silencing therapies such as short interfering RNA (69, 70). Among these, MYBPC3 gene replacement therapy is indicated to improve severe HCM myocytes via adeno-associated viral vector transfer (71). Another emerging targeted therapy is the small molecule myosin ATPase inhibitor (MYK-461), which shows the potential to reduce and prevent the development of HCM pathology including LVH, myocyte disorders, and cardiac fibrosis (72).

#### 4.3.3 Brugada syndrome (BrS)

BrS is an arrhythmic disorder associated with sudden death in young adults, the genetic background is the main determinant for the extent of the electrophysiological abnormalities. The only gene unequivocally associated with BrS is SCN5A. Patients carrying SCN5A mutations exhibited a spontaneous type 1 pattern and experienced more severe symptoms than the other subjects. Cardiac arrest, spontaneous life-threatening, and syncopal episodes were more frequent in SCN5A mutation-positive patients than in SCN5A mutation-negative patients (73, 74). The SCN5A coding sequence is too large to be cloned into adeno-associated virus (AAV) vectors, researchers have devised a gene therapy approach targeting MOG1, a chaperone protein involved in Na transport. TheAAV9-Mog1 gene therapy may improve patient prognosis by reducing the incidence of heart block, sinus arrest, and sinus arrhythmia (75). There are other genes associated with the prognosis of BrS patients. A recent study found that BrS patients with GSTM3 deletion presented more frequently with SCA than patients without GSTM3 deletion (76, 77), and there were more severe clinical presentations in GSTM3 deletion patients. These findings may provide information for subsequent stratified management of BrS patients (78, 79).

#### 4.3.4 Cardiac amyloidosis

Cardiac amyloidosis (CA) can be attributed to rare genetic variants or acquired diseases, and there are two main subtypes of the disease, transthyretin cardiac amyloidosis (ATTR-CA) and immunoglobulin light chain cardiac amyloidosis (AL-CA), which are characterized by the nature of the infiltrating proteins. ATTR-CA is further subdivided according to the presence or absence of mutations in the transthyretin genes into wild-type (ATTRwt-CA) and variant (ATTRv-CA) (80). ATTR-CA is highly associated with genetics due to the TTR gene variation. Until 2022, tafamidis is the only drug approved by the U.S. Food and Drug Administration (FDA) for the treatment of ATTR myocardial amyloidosis, but the high price limited the application (81, 82). Current studies have shown that small interfering RNA (siRNA) and antisense oligonucleotide (ASO) technologies are very effective in blocking TTR expression in the human liver. siRNA Patisiran and ASO inotersen have been approved for the therapy of patients with ATTR-mutant polyneuropathy, regardless of the presence and severity of ATTR cardiomyopathy. Preliminary data suggest that therapy with patisiran improves the cardiac phenotype in patients with ATTR variant polyneuropathy (83). In addition, another type of siRNA (vutrisiran) and a new ASO agent (eplontersen) will also be evaluated in Phase III clinical trials in patients with ATTR variant polyneuropathy or ATTR cardiomyopathy (84).

#### 4.3.5 Fabry disease (FD)

FD is caused by pathogenic variants in the GLA gene located on chromosome X. More than 1,000 variants distributed across the GLA gene have been identified. FD affects multiple systems, with cardiac involvement characteristics by progressive cardiac hypertrophy, fibrosis, arrhythmias, heart failure, and sudden cardiac death. Bidirectional sequencing of the seven coding exons (Sanger) and the exon-intron boundary of GLA are the gold standard for molecular diagnosis (85). Although FD was initially thought to predominantly affect males, recent studies have found that heterozygous Fabry females carrying a single mutant GLA gene can exhibit a wide range of clinical symptoms, challenging the notion of asymptomatic carriers (86). To understand the effect of different GLA mutations on the protein structure of *α*-galactosidase A, Li et al. (87) analyzed four GLA mutations using a bioinformatics approach and showed that these mutations not only have a significant effect on the internal dynamics and structure of GLA, but also lead to a significant reduction in enzyme activity. This finding provides a scientific basis for accurate diagnosis and precise medical intervention in FD (87). Further, Nagree et al. (88) showed that *in vitro* lentivirus-modified rapamycin-conditioned CD4 T cells can be propagated as a corrective enzyme for a variety of lysosomal storage disorders, including FD. The management of FD has undergone significant changes over the past two decades. Symptomatic treatment has shifted to a more integrative approach using FD-specific therapies, including the approved and currently available enzyme replacement therapy (ERT) and chaperone therapy (89–91). The most common therapy is enzyme replacement therapy with agatharesidase *α* and *β*, or the pharmacological oral chaperone Migalastat. These approaches can prevent the various complications of FD and ultimately alter the natural course of the disease (92).

### 4.4 Frontier research

HHD is a diverse and complex group of diseases, and many of the disease types are rare. Although we have summarized the latest research trends in several categories of HHD. Actually, the diagnosis and treatment of HHD are evolving faster than we think. In arrhythmogenic cardiomyopathies, several countries and teams have embarked research on the therapeutic potential of PKP2 gene replacement therapy (93). Current research projects include the Adeno-associated virus serotype 9 (AAV9): PKP2 gene therapy project, the TN-401 AAV9-Based Gene Therapy for PKP2-Associated Arrhythmogenic Right Ventricular Cardiomyopathy (ARVC), etc. These treatments prevent ventricular remodeling, delay the decline in left ventricular function, and reduce the incidence of arrhythmias. In the treatment of Brugada syndrome, Adeno-associated virus serotype rh.10 encoding for the human PKP2 gene (AAVrh.10hPKP2) therapy, which is being tested in multiple clinical trials in the United States in 2024. It offers a potentially one-time, curative treatment that promises to significantly improve survival and quality of patients’ lives. In Fabry disease therapy, the researchers investigated adeno-associated virus serotype 2/6 encoding human alpha-galactosidase A cDNA (AAV2/6hGLA), an adeno-associated virus (AAV)-based gene therapy vector recently (94). This vector encodes the human alpha-galactosidase A (GLA) gene and could provide a potential long-term treatment option for patients with Fabry disease that may be superior to existing enzyme replacement therapy (ERT). Further, recombinant adeno-associated virus vector serotype 5 (AAV5) harboring a codon-optimized human GLA transgene (AAV5-GLA) currently exhibits potent, long-lasting activity of GLA expression in animal models, and the favorable safety profile of adeno-associated virus gene therapy in humans has the potential to be a key research area for future clinical trials (95). For the frontier research, checking out some relevant registries may help to learn more advanced innovative research or trials, such as Orphanet, Union Register of Medicinal Products by European Commission, and U.S. Food and Drug Administration, et al.

### 4.5 Limitation

Our study has some limitations. First of all, it only uses the WoSCC database, and did not cover other public and commercial bibliometric databases such as PubMed, Scopus, Medline, and CNKI. Therefore, the data may not be comprehensive. However, this is mainly a limitation of the CiteSpace software itself. The citation and cited information in scientific texts reflect the flow and dissemination of scientific knowledge, which is an important theoretical basis for knowledge mapping. WoSCC is the most representative and widely used database in this field. Therefore, even if we only analyzed the literature from WOSCC, the findings remain reliable. Furthermore, some overlap may occur when analyzing the co-occurrence and clustering of keywords due to the presence of multiple synonyms. For these reasons, the literature search in this study is not comprehensive and may cause bias in the results.

## 5 Conclusion

To our knowledge, this is the first study to analyze and summarize the global research of genetic diagnosis and therapy of HHD by CiteSpace. Arrhythmogenic cardiomyopathy, hypertrophic cardiomyopathy, Brugada syndrome, Fabry disease, and cardiac amyloidosis are the most common HHD diseases. As for the genetic diagnosis and therapy of HHD, current research is not only pursuing the expansion of research cohorts and the innovation of diagnostic technology but also the wide application of gene therapy. Moreover, multidisciplinary management is the key direction in the future, and research in the genetic diagnosis and therapy of HHD should be broader, deeper, and more meticulous.
