# Supplementary material for: Advances in genetic diagnosis and therapy of hereditary heart disease: a bibliometric review from 2004 to 2024
Source: Front Med (Lausanne). 2025 Jan 8;11:1507313. doi: 10.3389/fmed.2024.1507313 (PMC11750821; doi:10.3389/fmed.2024.1507313)
Supplement: Supplementary file 1 [file Table_1.docx]

TABLE S1. Top 10 countries of studies on genetic diagnosis and therapy of hereditary heart disease (HHD).

| Rank | Country | Centrality | Count | Publications (%) |
| --- | --- | --- | --- | --- |
| 1 | USA | 0.02 | 1442 | 25.04 |
| 2 | ITALY | 0.05 | 566 | 9.83 |
| 3 | CHINA | 0.01 | 497 | 8.63 |
| **4** | ENGLAND | 0.11 | 443 | 7.69 |
| 5 | GERMANY | 0.04 | 364 | 6.32 |
| 6 | FRANCE | 0.10 | 250 | 4.34 |
| 7 | CANADA | 0.07 | 249 | 4.33 |
| 8 | NETHERLANDS | 0.09 | 247 | 4.29 |
| 9 | SPAIN | 0.16 | 243 | 4.22 |
| 10 | AUSTRALIA | 0.04 | 175 | 3.04 |
