# Supplementary material for: Advances in genetic diagnosis and therapy of hereditary heart disease: a bibliometric review from 2004 to 2024
Source: Front Med (Lausanne). 2025 Jan 8;11:1507313. doi: 10.3389/fmed.2024.1507313 (PMC11750821; doi:10.3389/fmed.2024.1507313)
Supplement: Supplementary file 2 [file Table_2.docx]

TABLE S2. Top 10 institutions of studies on genetic diagnosis and therapy of hereditary heart disease (HHD).

| Rank | Institution | Country | Centrality | Count |
| --- | --- | --- | --- | --- |
| 1 | Harvard University | USA | 0.1 | 202 |
| 2 | University of London | ENGLAND | 0.11 | 192 |
| 3 | Assistance Publique Hopitaux Paris (APHP) | FRANCE | 0.19 | 145 |
| **4** | University College London | ENGLAND | 0.09 | 139 |
| 5 | Mayo Clinic | USA | 0.05 | 137 |
| 6 | Harvard Medical School | USA | 0.04 | 127 |
| 7 | Institut National de la Sante et de la Recherche Medicale (Inserm) | FRANCE | 0.1 | 120 |
| 8 | University of California System | USA | 0.12 | 117 |
| 9 | University of Pennsylvania | USA | 0.07 | 96 |
| 10 | University of Amsterdam | NETHERLAND | 0.05 | 89 |
